# Supplementary material for: Aquatic Biomaterial Repositories: Comprehensive Guidelines, Recommendations, and Best Practices for Their Development, Establishment, and Sustainable Operation
Source: Mar Drugs. 2024 Sep 20;22(9):427. doi: 10.3390/md22090427 (PMC11433314; doi:10.3390/md22090427)
Supplement: Supplementary file 1 [file marinedrugs-22-00427-s001.zip › marinedrugs-3160243-supplementary.pdf]

Aquatic Biomaterial Repositories: Comprehensive guidelines, recommendations and best practices for their development, establishment and sustainable operation

Christiana Tourapi [1], Eleni Christoforou [1], Susana P. Gaudêncio [2,3] and Marlen I. Vasquez [1*]

[1] Department of Chemical Engineering, Cyprus University of Technology, Archiepiskopou Kyprianou 30, 3036 Limassol, Cyprus; ctourapi@yahoo.com; eleni.christoforou@cut.ac.cy

[2] Associate Laboratory i4HB, Institute for Health and Bioeconomy, NOVA Faculty of Sciences and Technology, NOVA University of Lisbon, 2819-516 Lisbon, Portugal; s.gaudencio@fct.unl.pt

[3] UCIBIO, Applied Molecular Biosciences Unit, Chemistry Department, NOVA Faculty of Sciences and Technology, NOVA University of Lisbon, 2819-516 Lisbon, Portugal

* Correspondence: marlen.vasquez@cut.ac.cy; Tel.: +35799332463

Table S1: List of abbreviations.

Abbreviation	Definition
ABCD	Access to Biological Collection Data
ABRs	Aquatic Biomaterial Repositories
ABS	Access and Benefit Sharing
BEBs	Biodiversity and environmental biobanks
BLAST	Basic Local Alignment Search Tool
BRISQ	Biospecimen Reporting for Improved Study Quality
BUILD	Boosting Uganda's Investment in Livestock Development
CBD	Convention on Biological Diversity
cDNA	copy/complementary DNA
CITES	Convention on International Trade in Endangered Species of Wild Fauna and Flora
CMS	Conservation of Migratory Species
DNA	Deoxyribonucleic acid
DwC	Darwin Core
EMBRC	European Marine Biological Resource Centre
EMFF	European Maritime and Fisheries Fund
EMO BON	European Marine Omics Biodiversity Observation Network
ESBs	Environmental Specimen Banks
EU	European Union
GES	Good environmental status
GGBN	Global Genome Biodiversity Network
GGI	Global Genome Initiative
GSC	Genomic Standards Consortium
INSDC	International Nucleotide Sequence Database Collaboration
ISBER	Society for Biological and Environmental Repositories
IUCN	International Union for Conservation of Nature
LIMS	Laboratory Information Management System
LMOs	Living modified organisms
MIGS	Minimum Information about a Genome Sequence
MIMARKS	Minimum Information about a MARKer gene
MSFD	Marine Strategy Framework Directive
NGS	Next-generation sequencing
NHC	Natural History Collections
NIST	National Institute of Standards and Technology
NMMTB	National Marine Mammal Tissue Bank
NOAA	National Oceanic and Atmospheric Administration
NP	Natural products
PCR	Polymerase chain reaction
RNA	Ribonucleic acid
RRI	Responsible Research and Innovation
SOPs	Standard Operating Procedure
SPREC	Standard PRE analytical Codes
UNCLOS	United Nations Convention on the Law of the Sea
WFD	Water Framework Directive

WTO	World Trade Organisation

Table S2: Examples of Marine Environmental Biospecimen Repositories.

**Marine Environmental Biospecimen Repositories**	**References**
**1-2:** The National Institute of Standards and Technology (**NIST**)<br>**3:** International Society for Biological and Environmental Repositories (**ISBER**)<br>**4:** Boosting Uganda's Investment in Livestock Development (**BUILD**)<br>**5-6:** The European Marine Biological Resource Centre (**EMBRC**<br>**7:** The **NIST** Biorepository (formerly known as the Marine Environmental Specimen Bank)<br>**8:** National Marine Mammal Tissue Bank (**NMMTB**) - NOAA Fisheries<br>**9:** Global Genome Initiative (**GGI**) - Smithsonian National Museum of Natural History<br>**10:** National Marine Biodiscovery Laboratory - Biomaterials Repository - Marine Institute **EMFF** 2014 – 2020<br>**11:** Marine Institute **Foras na Mara**	**1.** Pugh R.S., Becker P.R., Porter B.J., Ellisor M.B., Moors A.J., Wise S.A. (2008) Design and Applications of the National Institute of Standards and Technology's (NIST's) Environmental Specimen Banking Programs. CELL PRESERVATION TECHNOLOGY. Volume 6, Number 1. https://doi.org/10.1089/cpt.2007.0517<br>**2.** Becker P.R., Gunter E.W., Schluter C., Shibata Y., Wise S.A. (2006). Environmental specimen banking. J. Environ. Monit., 8, 776–778. https://doi.org/10.1039/b608753c<br>**3.** BEST PRACTICES: Recommendations for Repositories, Fourth Edition. (2018). ISBER, Canada. www.isber.org<br>**4.** Mugizi, D., Obilil, I. and Roesel, K. 2020. BUILD biorepository workflows. Poster prepared for the Virtual Annual Planning Meeting ILRI/BMZ Program, Boosting Uganda's Investment for Livestock Development (BUILD), 10–12 June 2020. Nairobi, Kenya: ILRI. https://hdl.handle.net/10568/109524<br>**5.**https://www.embrc.eu/<br>**6.**Nardello, Ilaria and Villanueva, Antonio and Kloareg, Bernard and Kooistra, Wiebe and Merciecca, Matthieu. (2017). EMBRC-ERIC Business Plan, Update 2017. https://www.researchgate.net/publication/327338359_EMBRC-ERIC_Business_Plan_Update_2017.<br>**7.**https://www.nist.gov/programs-projects/nist-biorepository<br>**8.**https://www.fisheries.noaa.gov/national/marine-mammal-protection/national-marine-mammal-tissue-bank<br>**9.**https://naturalhistory.si.edu/research/global-genome-initiative<br>**10.**https://emff.marine.ie/<br>**11.**https://www.marine.ie/
